# A citizen scientist’s survey provides insights into the movements of Atlantic and Gulf states’ populations of Black Skimmers (*Rynchops niger*)

**DOI:** 10.1371/journal.pone.0347650

**Published:** 2026-05-06

**Authors:** Brett R. Moyer

**Affiliations:** Science Department, The Bolles School, Jacksonville, Florida, United States of America; MARE – Marine and Environmental Sciences Centre, PORTUGAL

## Abstract

Ornithologists are increasingly using field readable bands—plastic rings with alphanumeric codes that are visible from a distance—to study bird populations. Because of this advance, a member of the public can now readily engage in research. On one level, such a citizen scientist can report hundreds of sightings to help the banding scientists answer their research questions. On another level, the uniquely marked birds create opportunities for that citizen scientist to conduct independent research that offers a novel perspective. I describe the results of a five-year study in Florida where I photographed 1041 bands, representing 347 Black Skimmer (*Rynchops niger*) individuals. I show that skimmers travel to Northeast Florida in large numbers from Atlantic states up to 1500 km away. In contrast, movement to NE Florida from populations along the Gulf of Mexico was limited. My encounter rate of birds from the more distant Atlantic states was nearly 20x higher than birds from Western Florida, which were as close as 275 km away. I discuss why the birds travel to NE Florida, ranging from overwintering, to staging during migration, to dispersal for breeding. I report the longest natal dispersal (281 km) for the Black Skimmer, documenting gene flow between the Gulf and Atlantic populations. The approach modeled here could be applied by citizen scientists investigating other bird species and non-avian species with field readable tags.

## Introduction

Citizen science, defined as “public participation in scientific research and knowledge production,” is growing, particularly in fields of environment science and ecology [1,2]. These non-professionals can enhance research by contributing large numbers of observations across vast geographic scales, allowing investigations that the scientists who study wide-ranging species would not have the time or resources to do themselves [1]. Moreover, citizen scientists themselves can make discoveries when they contribute their local knowledge or unique perspectives to interactive online forums [3].

One way to participate as a citizen scientist in ornithology is to report observations of banded birds. The United States Geological Survey (USGS) Bird Banding Laboratory (BBL) has been collecting information on banded birds for over 100 years. Historically, data were compiled from hunters who found the uniquely numbered metal bands on their harvest, from people who found a dead banded bird in the wild, and from scientists who captured and released previously banded birds. Although the frequency that any individual metal band was resighted was low, the collective effort allowed information to funnel back to the scientists doing the original banding, and that has provided insights into the movement and conservation of various bird species [4,5].

The introduction of field readable bands over the last few decades has transformed mark and recapture studies with birds. The metal band used as the previous standard was stamped with small text that could be reliably read only if the bird was in hand. Conversely, field readable bands are colored plastic and have an alphanumeric code etched in a large font of contrasting color, which allows them to be viewed at a distance. This advance has greatly increased the resighting rates for individual birds [6].

Historically, a citizen scientist might have had a chance discovery of a dead bird that had a metal band, they reported that band, and they received information from the BBL that gave data about that bird. With field readable bands, however, what was once an isolated data point can now potentially become hundreds or even thousands of data points. This has expanded opportunities for citizen scientists to help professional scientists collect data to answer their research questions on wide-ranging species.

Citizen scientists have made massive contributions in ornithology via such big data networks such as eBird [7]. On one hand, technological advances now allow university researchers to analyze millions of datapoints submitted by hundreds of thousands of citizen scientists [7]. The present article will explore the other hand—how advances now allow individual citizen scientists to conduct their own lines of inquiry. The existence of the uniquely marked birds allows members of the public to conduct independent research that offers a novel perspective.

Black Skimmers (*Rynchops niger*) provide an opportunity for citizen scientists to conduct research to benefit an imperiled species, as populations of this species are of special concern in multiple US states, including being designated as threatened in the state of Florida [8,9]. If one examines the distribution map of Black Skimmers in the eastern United States, one sees a continuous range along the coast, spanning from North Atlantic states to the Western Gulf of Mexico. There is little published information regarding the movement of Black Skimmers within this range [9,10]. Researchers in ten states along the US coast have been fitting Black Skimmers with field readable bands since 2015 [11]. The centralized geographic location of NE Florida provides a strategic vantage point to investigate the connectivity of the Atlantic coast populations, which stretch 1500 km to the North to Massachusetts, and the Gulf coast populations, which stretch 1300 km to West to Texas.

Here, I report 1041 sightings of field readable bands on Black Skimmers in Northeast Florida, representing 347 unique individuals. I show how these data provide information regarding the migration and dispersal of birds among Gulf and Atlantic States of the USA.

## Materials and methods

From 23 January 2021 to 16 July 2025, I visited public beach parks in Northeast Florida 132 times to look for Black Skimmers with field readable bands. Visits occurred in all 12 months of the year but were concentrated from November-March (Table 1). I visited Huguenot Memorial Park (GPS: 30.40613, −81.40399)(n = 109 visits), Amelia Island State Park (30.51515, −81.43815) (n = 7), Anastasia State Park (29.90486, −81.27811) (n = 9), Big Talbot Island State Park (30.51160, −81.45621) (n = 2), Fort Clinch State Park (30.70028, −81.42632) (n = 2), Fort Matanzas National Monument (29.70590, −81.22958) (n = 2), and Peters Point Beachfront Park (30.59990, −81.44233)(n = 1).

**Table 1:** The number of surveys at beach parks in NE Florida, by month, from January 2021 to July 2025.

	Aug	Sep	Oct	Nov	Dec	Jan	Feb	Mar	Apr	May	Jun	Jul
**n**	**1**	**2**	**9**	**18**	**19**	**17**	**22**	**16**	**7**	**8**	**12**	**1**

**Fig 1:**
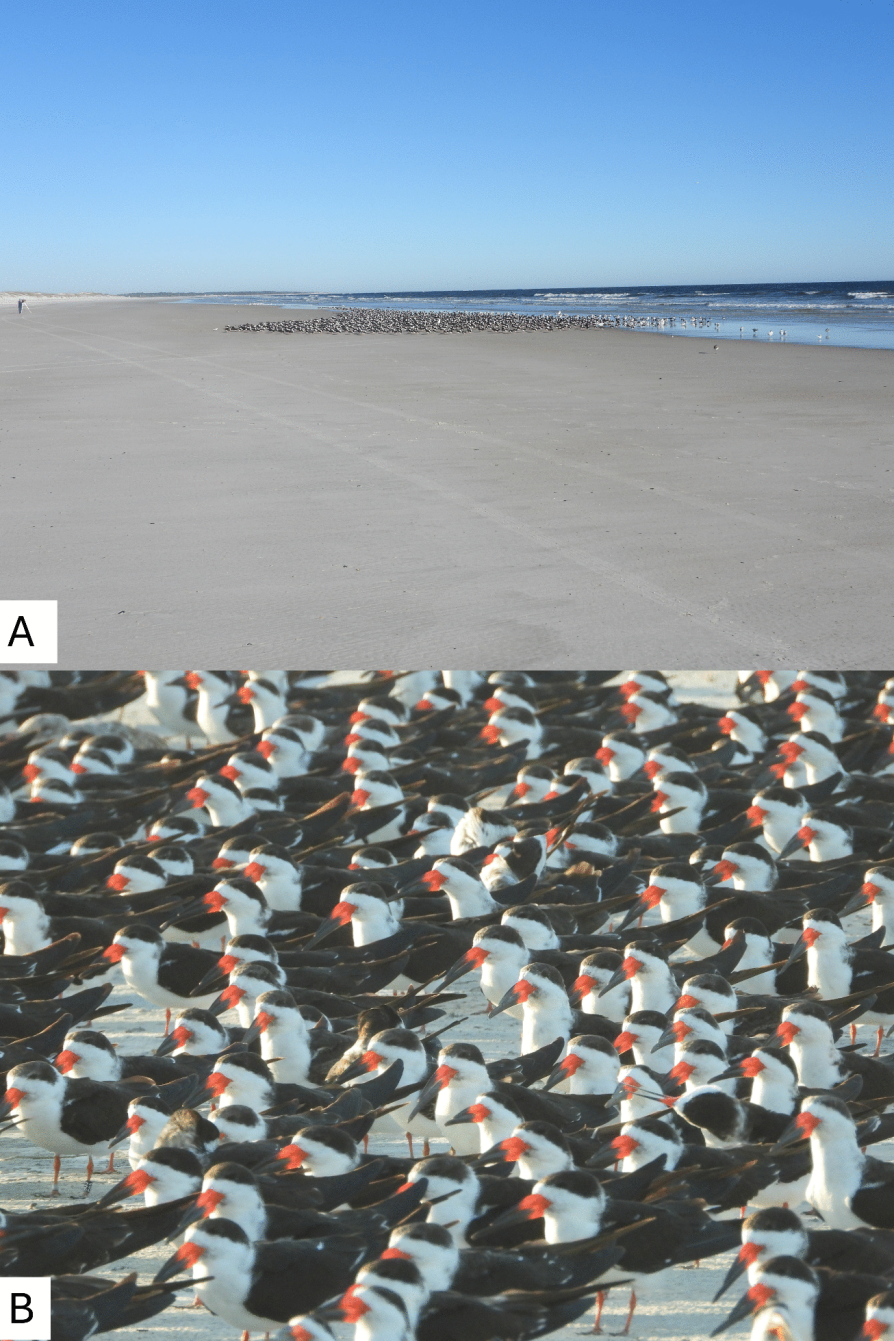
Black Skimmers resting on the beach at Huguenot Memorial Park. **(A)** Shows a flock of about 1000 Black Skimmers resting on the beach on 28 December 2022. On this day I was able to photograph 51 unique bands. Note that an observer with a spotting scope is visible for scale in the left side of the photograph. **(B)** Shows a portion of a flock of Black Skimmers resting on the beach at Huguenot Memorial Park on 4 February 2023. Note the bird with the field readable band, Black C05, in bottom left of the photograph.

I examined resting flocks of Black Skimmers (Fig 1) from a distance and photographed any field readable bands using a Nikon P1000 superzoom digital camera, which has a 125x optical zoom (24–3000 mm equivalent). As such, no research permits were required, as the birds were on a public beach and I was among the fishermen, surfers, birdwatchers and other beachgoers present. I accessed no restricted areas. Visits could last up to six hours at locations that required hiking to different sections of the beach and on days when there were large numbers of birds to observe and photograph. Some visits were less than an hour if it was a small park and there were few birds present on that day.

Each band code observed was reported to the Federal Bird Banding Lab (www.reportband.gov). Photographs of banded birds were saved and photos of any bands that were new to me were uploaded (using the username “Bluebird Pharmacy”) to the Facebook Group, “Florida Banded Bird Resightings,” where I also presented a complete list of all the bands I observed on that day.

The Bird Banding Lab would email a “Certificate of Appreciation” following the band report. This document provides various data for each bird, including such information as the location and date that the bird was originally banded and the name of the banding scientist. I entered the data from these certificates into a spreadsheet. Through correspondence with various scientists, I was able to determine the total number of field readable bands that were fitted to Black Skimmers in each state by the end of the 2024 field season. No Black Skimmers are currently banded in NE Florida, and no birds banded in other regions during the first few months (June/July) of the 2025 field season were observed in NE Florida prior to the completion of the study in July 2025, as they hadn’t yet left their natal colony. Accordingly, the number of birds banded by the end of the 2024 season is the number of banded birds that could be observed in NE Florida through July 2025.

To test whether the Black Skimmer populations to the north (Atlantic: MA, NY, NJ, VA, NC, SC) and to the west (Gulf: FL, MS, LA, TX) were represented equally in NE Florida, I performed a chi-square test of independence comparing the proportion of individuals of the Atlantic and Gulf populations observed in NE Florida versus not observed. A 2 x 2 contingency table was constructed using observed counts, and expected values were calculated under the assumption of equal proportional representation across populations. I conducted a second analysis with the Gulf population limited to FL (excluding the more distant MS, LA, and TX) to test whether the Black Skimmer populations to the north (Atlantic: MA, NY, NJ, VA, NC, SC) and to the west (Gulf: FL) were represented equally in NE Florida. (Note: no skimmers from the Atlantic coast of Florida have been banded yet).

Natal dispersal distance was measured by drawing a straight line between the GPS location of the colony where the bird was banded as too young to fly—as derived from the data on record at the USGS Bird Banding Lab—and the GPS coordinates of the colony where the bird was recorded breeding in the present study, and then using the distance calculator at www.calculator.net/distance-calculator.html.

## Results

**Fig 2:**
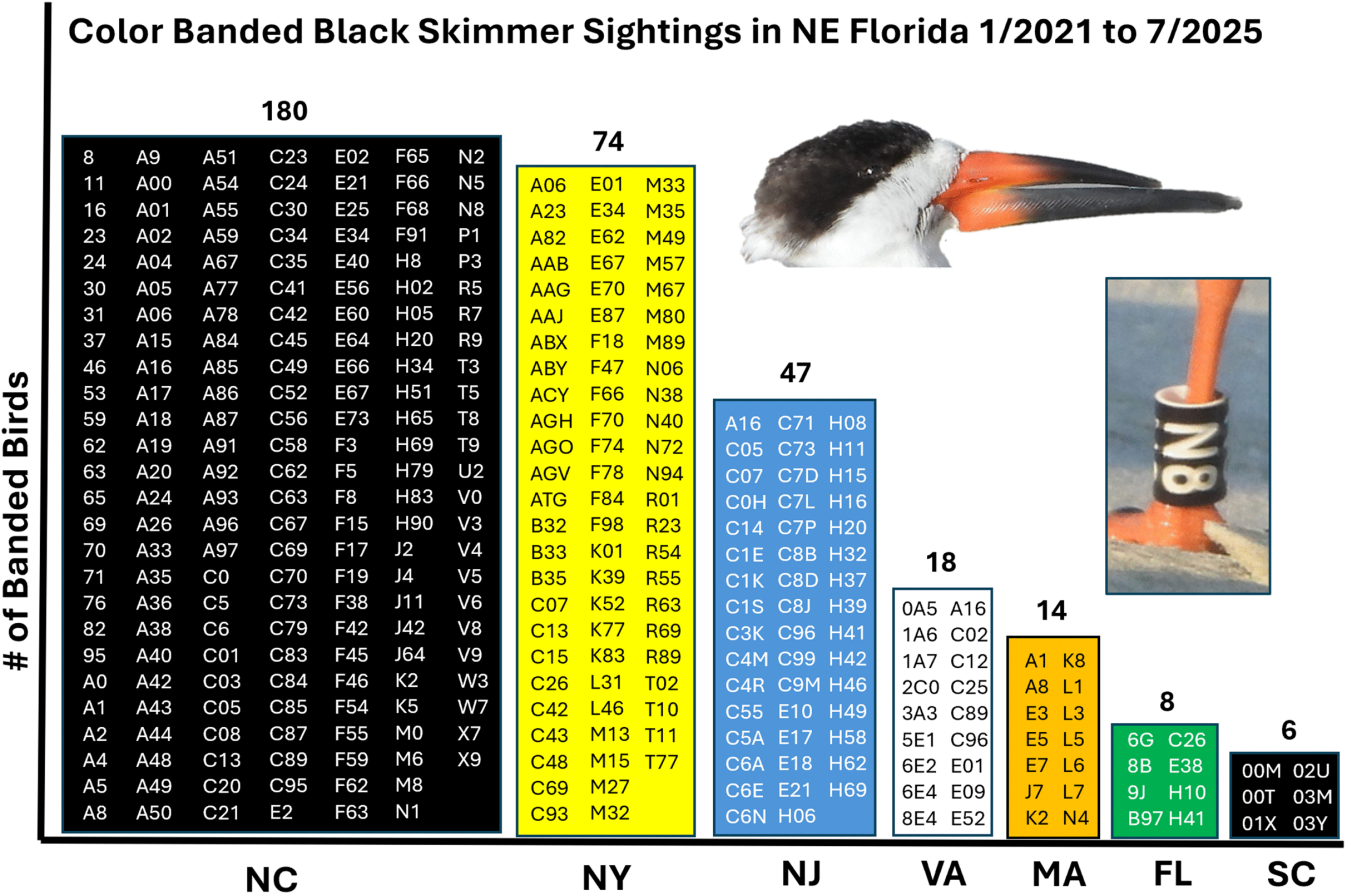
Band codes for Black Skimmers observed in Northeast Florida. A summary of the 347 unique band codes for Black Skimmers that I observed in NE Florida from January 2021 to July 2025. The graph shows the number of birds from each state and the code of the field readable band for each. The color of the column represents the color of the band for that state. Note that NC and SC birds both have black bands, but NC birds have black bands on the left leg with white codes that are #, ##, alpha#, and alpha## (e.g., Black N8 is alpha#). SC birds have black bands on the right leg with white codes that are ##alpha (e.g., Black 00M). The abbreviations for US states are: Massachusetts (MA), New York (NY), New Jersey (NJ), Virginia (VA), North Carolina (NC), South Carolina (SC), Florida (FL).

**Fig 3:**
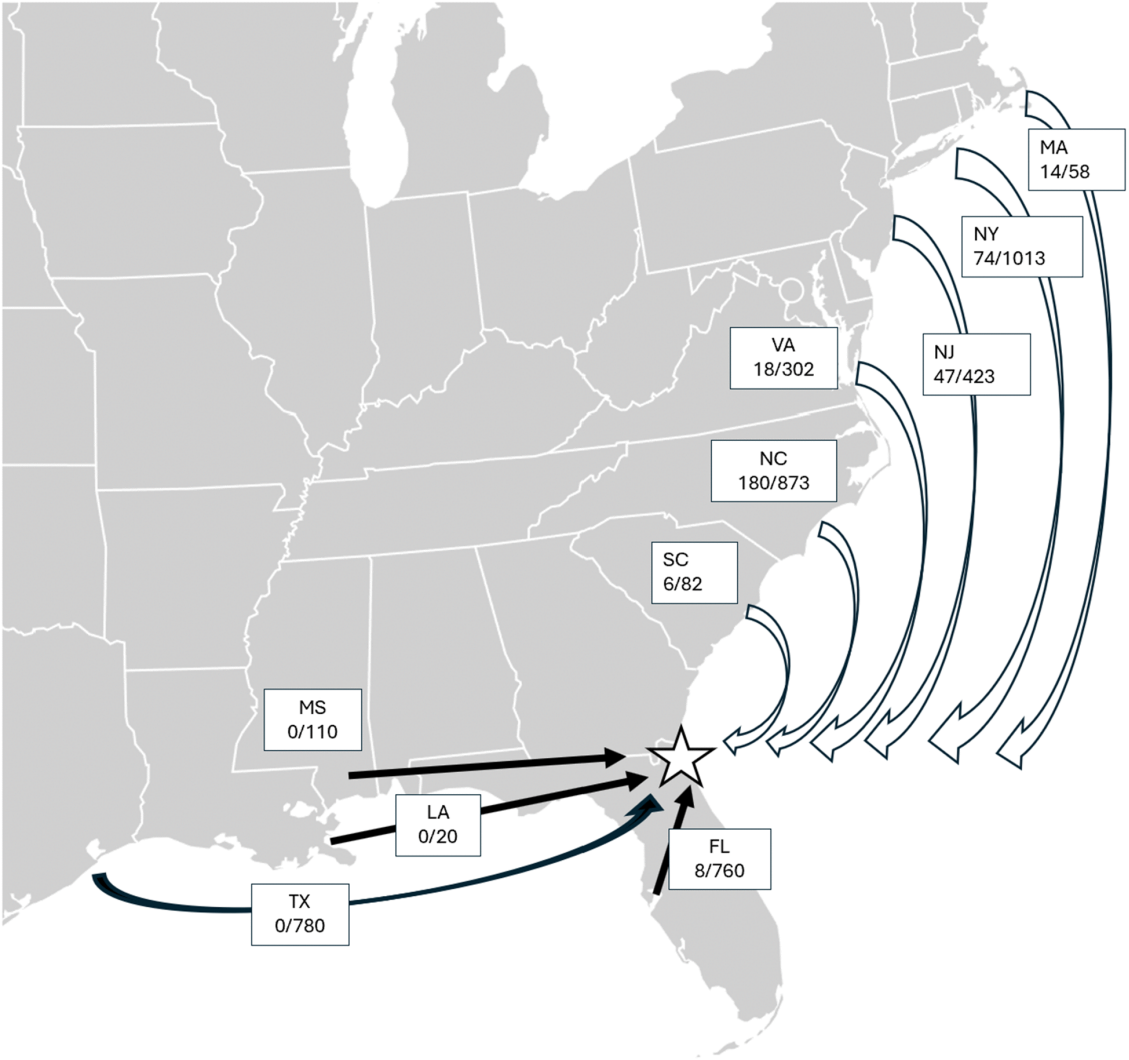
Movement of Black Skimmers from different states to NE Florida. The denominator for each state is the total number of field readable bands that had been fitted on Black Skimmers in that state through the completion of the 2024 field season. The numerator is the number of unique band codes that I have observed from that state in NE Florida through July 2025. White arrows are used for populations along the Atlantic coast to the north of the study site, and solid black arrows are used for populations along the Gulf of Mexico to the west of the study site. Modification of base map with CC0 designation from https://en.wikipedia.org/wiki/File:Blank_US_Map_(states_only).svg.

Over the four and a half years of field observation, 1041 Black Skimmers with field readable bands were observed in Northeast Florda, representing 347 unique individuals from seven states (Fig 2). Overall, the encounter rate of Atlantic coast populations of Black Skimmers in NE Florida was high, in contrast to that of Gulf State populations (Fig 3)

Banded individuals from populations to the north (Atlantic: MA, NY, NJ, VA, NC, SC) were observed in NE Florida significantly more frequently than populations to the west (Gulf: FL, Mississippi (MS), Louisiana (LA), and Texas (TX) were observed in NE Florida (12.3% vs 0.48%). A chi-square test revealed a highly significant departure from equal representations. (χ^2^ = 199.9, df = 1, *p* < 0.001). Banded individuals from populations to the north (Atlantic: MA, NY, NJ, VA, NC, SC) were observed in NE Florida significantly more frequently than populations from Florida were observed in NE Florida (12.3% vs 1.05%)(χ ^2^ = 83.6, df = 1, *p* < 0.001).

At least one Black Skimmer band code was recorded during 106 of the 132 visits to the beach parks. Table 2 highlights the ten days with the highest number of different field readable band codes observed—all were at Huguenot Memorial Park. The highest number documented on a single day was 51 unique codes on 28 December 2022 from a flock of over 1000 individuals (Fig 1A).

**Table 2:** The visits to beach parks in NE Florida in which the highest number of unique band codes were observed. All records in this top ten were from Huguenot Memorial Park.

Date	Number of different band codes observed
28 Dec 2022	51
27 Jan 2024	45
9 Mar 2024	45
15 Jan 2023	32
31 Dec 2023	31
3 Feb 2024	28
24 Feb 2024	28
14 Dec 2024	27
8 Mar 2025	26
10 Mar 2025	26

**Fig 4:**
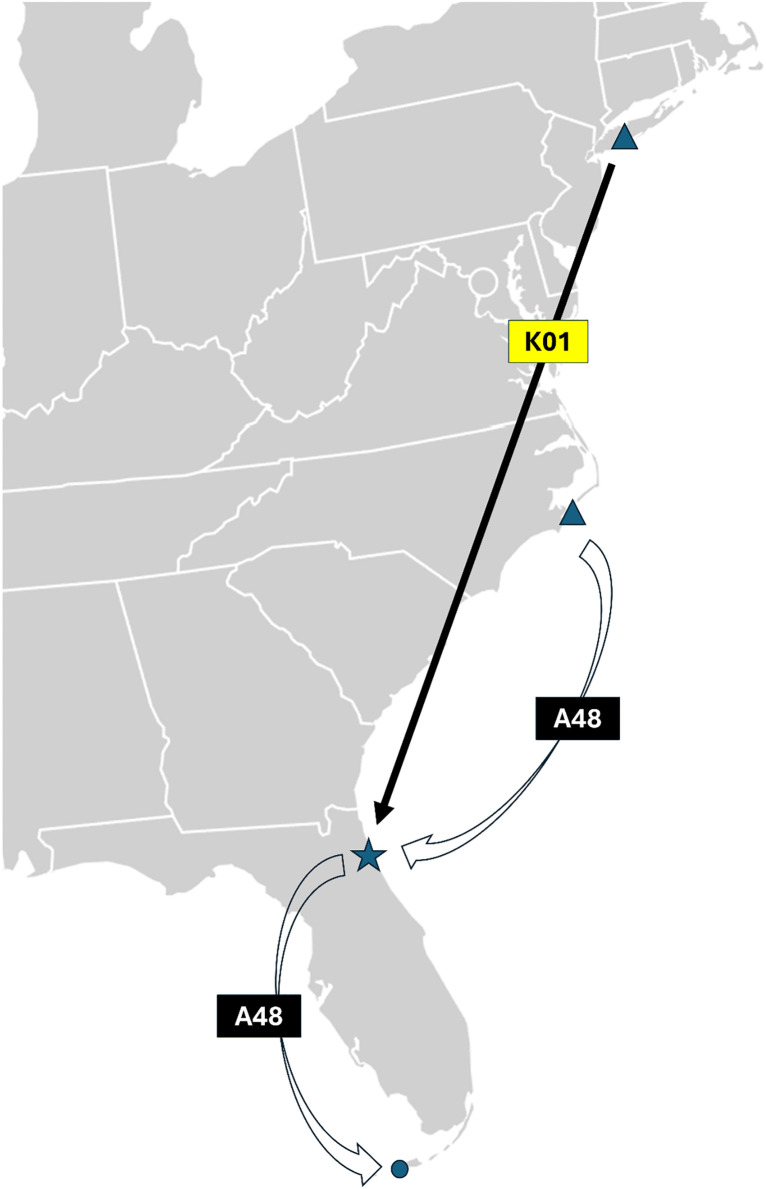
Path of a Black Skimmer using NE Florida as wintering grounds (band code Yellow K01), versus one using NE Florida as staging grounds (Black A48). Both birds were observed at Huguenot Memorial Park (blue star). Yellow K01 is originally from New York. Black A48 was originally banded in North Carolina and passed through Huguenot on its way to Key West, Florida (blue circle). Modification of base map with CC0 designation from https://en.wikipedia.org/wiki/File:Blank_US_Map_(states_only).svg.

Yellow K01, originally banded in New York in 2020, appears to be using Northeast Florida as overwintering grounds. I observed it 41 times, spread across every year of the study, arriving as early as mid-September and departing as late as mid-April (Fig 4)

Black A48, originally banded in North Carolina in 2021, is using NE Florida as a stopover site during its migration further south (Fig 4). I saw it in Northeast Florida only three times (12 February 2022, 18 November 2023, and 10 March 2025) over four non-breeding seasons. The story becomes more complete, however, when one consults posts by another citizen scientist (Ingrid Siegert) on the Florida Banded Bird Resightings group on Facebook. The data from the last two years is instructive. I saw Black A48 in November 2023 as it was passing through NE Florida. It was then observed many times 600 km to the south in Key West, FL in Jan-Feb of 2024. It was observed the following season again in Key West from December 2024 to February 2025. It was last recorded in Key West FL on 9 February 2025, and I saw it in NE Florida on 10 March 2025. Thus, from the observations of just two citizen scientists, it appears that Black A48 passes through NE Florida in mid-November, spends late December to early February in Key West and then passes back through NE Florida in mid-February to early March on its way back up North.

**Fig 5:**
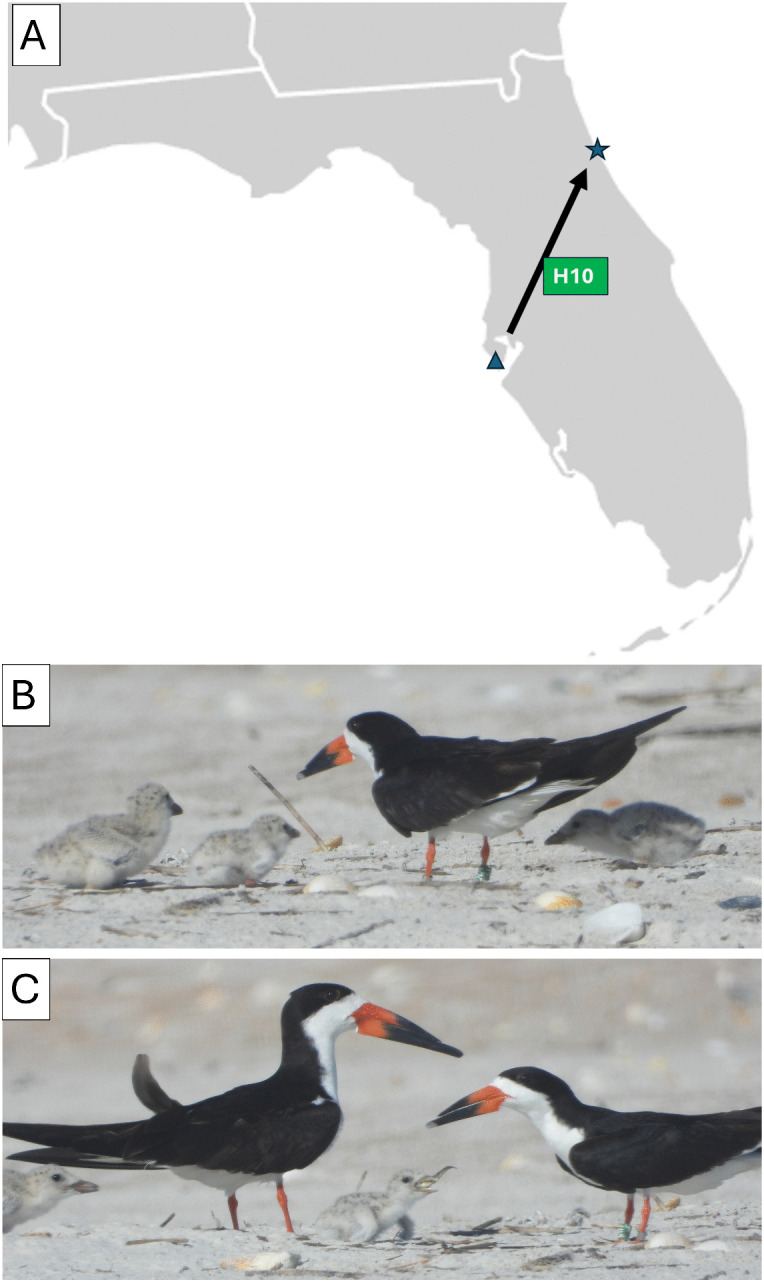
Natal dispersal of Black Skimmer with the band code Green H10. **(A)** The 281.3 km path from Pinellas Co., Florida, on the Gulf of Mexico where Green H10 was banded as too young to fly on 8 July 2023 (blue triangle) to Anastasia State Park in St. Johns County, FL where it was documented as breeding on 16 July 2025 (blue star). **(B)** Green H10 with its three young chicks staying close to it on 16 July 2025. **(C)** Green H10 moments after it was observed to return from a foraging run and feed a fish to one of its chicks. Note the fish that is still visible in the mouth of the chick. Modification of base map with CC0 designation from https://en.wikipedia.org/wiki/File:Blank_US_Map_(states_only).svg.

Green H10 dispersed from the colony in Pinellas County, FL where it was banded on 8 July 2023 as too young to fly (27.71833, −82.74032) to Anastasia State Park in St. Johns County, FL where it was recorded breeding (29.90595, −81.27962), a distance of 281.3 km (Fig 5A). On 16 July 2025 Green H10 was photographed with its three chicks huddled around it (Fig 5B) and shortly after it was observed feeding a chick (Fig 5C).

## Discussion

These findings show that Black Skimmer populations travel to Northeast Florida in large numbers from Atlantic states during the non-breeding season from as far away as Massachusetts, which is over 1500 km distant. In contrast, the results document quite limited movement to NE Florida for populations along the coast of the Gulf of Mexico, even from populations in Western Florida that are only 300 km distant (Fig 3).

The encounter rate of the field readable bands from Florida (Green bands) in Northeast Florida was only 1.1% (8/760), but the encounter rate of field readable bands from North Carolina (Black) was 20.6% (180/873) and Massachusetts was 24.1% (14/58) (Fig 3). Thus, birds from NC and MA were encountered in Florida at nearly 20x the rate of Florida birds in Florida. Currently, only Gulf Coast populations of Black Skimmers are being fitted with field readable bands in Florida, and this study shows that those birds travel infrequently to the study area in NE Florida, compared to birds from the Atlantic States. The lack of sightings of birds from Texas, Louisiana and Mississippi (0 of 910 birds seen), despite hundreds of hours of observations, also provides important information. Clearly, Black Skimmers from these three Gulf states do not regularly travel to NE Florida. A subspecies of Black Skimmer (*Rynchops niger cinerascens*) is known through telemetry studies to cross the Peruvian Andes at an elevation of nearly 5000 m in its travels between the Amazon and the western coast of South America [12]. Accordingly, a 300 km trip across inland Florida, where the highest elevation in the state is 105 m would not seem to be a challenging barrier.

There are at least three reasons that Black Skimmers travel to Northeast Florida: (1) overwintering, (2) as a stopover before continuing migration, and (3) dispersal for breeding. These cases are exemplified by three of the 347 banded individuals observed: Yellow K01, Black A48, and Green H10. For the overwintering (Yellow K01) and staging birds (Black A48) (Fig 4), the master bird banders who receive notifications of every encounter of a particular bird that is reported to the BBL will be able to map their journeys throughout the year in greater detail.

The dispersal of Green H10 to Northeast Florida to breed (Fig 5) warrants further discussion. Spicer and Forys [13] banded Black Skimmer chicks at several colonies in Southwest Florida and documented where they dispersed for their first breeding. The researchers found that only 4 of 86 birds (5%) dispersed more than 200 km, with 242.6 km being the farthest. Spicer and Forys [13] write, “Although southwest Florida supports the largest number of Black Skimmers in the state, there are colonies in northern Florida, and 14 of the 86 Black Skimmers in our study have spent time outside of the breeding season near some of these colonies, *but there is no record of them nesting.*” (emphasis mine) Accordingly, the natal dispersal of 281.3 km that I documented from one of their colonies to Anastasia State Park (Fig 5A-5C) is the longest distance in the published record for the Black Skimmer. It is also the first record of an individual dispersing from the southwest colonies to successfully breed in one of the colonies in the north of Florida (Gulf or Atlantic), and it is only the second record of natal dispersal to a colony on the Atlantic Coast of Florida—the only other being a bird that dispersed to breed at a rooftop colony at Patrick Airforce Base, which is located mid-state on the east coast of Florida [13].

Recent changes to the beach habitat appear to have facilitated this first documented natal dispersal to NE Florida. Prior to 2024, the beaches of Anastasia State Park had become critically eroded [14]. Two events improved beach conditions prior to the 2025 nesting season. First, a renourishment project by the US Army Corp of Engineers in September 2024 added nearly 2 million cubic meters of sand and extended into the southern end of the park, markedly expanding the beach [15]. Interestingly, the report from the US Army Corps of Engineers stated that, “The design intent of placing an extra-wide beach near the pier allows waves and currents to quickly reshape the fill by transporting sand from the dry beach and depositing it below the waterline and to the adjacent beaches north and south.” [15] Second, and consistent with the described plan, prior to the 2025 nesting season storms deposited sand and reshaped the beach at the northern end of the park, which is a 4.5 km hike from the main access point at the south of the park and has reduced human disturbance as a result [16]. The combined result was to increase the area of beach suitable for nesting shorebird species at the park during the 2025 season. Black Skimmers benefitted. Although Black Skimmer breeding activity had been minimal in prior years at Anastasia State Park, the colony they formed at the northern end of the park in 2025 resulted in a nesting year that was described as the “best Black Skimmer season in decades with more than 30 fledged young” [17].

Green H10 may not have been the only instance of natal dispersal to Anastasia State Park in 2025. I also recorded two other banded birds from SW Florida (Green H41 and Green B97) among the expanded population of skimmers present at the colony. Both individuals exhibited behaviors that suggested they might also be breeding, as they were observed standing near a chick for extended periods. Black Skimmer colonies can be crowded and bustling locations, however, so definitive evidence of breeding requires observing an individual incubating eggs or feeding a chick (evidence recorded for Green H10 in Fig 5C). Recall that only 8 of the 780 skimmers banded in SW Florida were observed in NE Florida during the five-year survey. That three of those eight were present at the Anastasia SP colony during both July and August of 2025 is intriguing. The Anastasia SP colony is one of only two ground nesting colonies for Black Skimmers along the more than 600 km of the Atlantic coast of Florida [16], and the only one in NE Florida. Given that there are estimated to be fewer than 7000 breeding Black Skimmers in Florida [18], dispersal and breeding of individuals from populations in SW Florida to NE Florida would have important conservation implication for this imperiled species [8,19].

Although the current paper shows that, overall, Gulf populations of Black Skimmers are relatively disconnected from the Atlantic coast, if beach conditions in subsequent years continue to support the expanded Anastasia State Park colony, that location may be an important site of gene flow between the Atlantic and Gulf populations. Goodenough et al [10] did not sample individuals in Florida in their study of population genetics of Black Skimmers, but they noted that the geographic location of these Florida residents offers an interesting opportunity to investigate gene flow in North American populations of skimmers.

The productive fisheries of NE Florida [20,21] are likely an important reason that Black Skimmers travel to the region for overwintering, staging, and breeding. Turtora and Schotman [21] conducted intensive sampling and identification of more than 350,000 fish and invertebrates in the coastal lagoons of northeast Florida. These habitats are part of the foraging grounds of the Black Skimmer, so their study provides a window into what food items are available to the birds. Of particular interest, one of Turtora and Schotman’s sampling sites was the St. Augustine Inlet, which forms the northern limit of Anastasia State Park and is a mere one kilometer from the breeding colony where Green H10 raised chicks. Of the 35,000 samples they took in the St. Augustine Inlet over several years, the most frequently taken fish genera were (in descending order): *Anchoa* (anchovy), *Leiostomus* (croaker), *Mugil* (mullet), *Menidia* (silverside), *Fundulus* (killifish), and *Eucinostomus* (mojarras) [21]. Black Skimmers are known to consume all these species [9,22]

Huguenot Memorial Park and Anastasia State Park are important for the conservation of Black Skimmers. These preserves are positioned amidst the dynamic estuarine ecosystem of NE Florida, where rivers, tidal lagoons, and extensive salt marshes interact to create a highly productive habitat [20,21,23] to support breeding and migrating individuals. Furthermore, the beaches and dunes in these two parks and others nearby provide one of the last undeveloped nesting habitats along the Atlantic Coast of Florida [23].

The research reported in this article would not have been possible before 2015, the year that scientists studying populations of Black Skimmers on the Gulf and Atlantic Coasts of the US started to use field readable bands [11]. The high count of Black Skimmers recorded on eBird for the entire United States was 8250 birds on 17 December 2009 [24], which was also at Huguenot Memorial Park, reinforcing this park’s importance for Black Skimmer conservation. I recorded 51 unique band codes in a flock of about 1000 skimmers at Huguenot on 28 December 2022. It is interesting to note, however, that if an army of citizen scientists had been diligently scanning that record aggregation of 8250 skimmers on 17 December 2009, they would have likely resighted zero bands, unless they happened to have found a dead skimmer with the USGS metal band that could be read in hand. The implementation of field readable bands with their visible alphanumeric codes has created new opportunities in citizen science.

The investigation described here contributes on two levels. On one level, I reported 1041 sightings to the Bird Banding Lab, so the fourteen scientists who banded those individuals received numerous updates from the BBL for the subset of birds they banded, giving them data to explore the focal questions of their respective research programs. A single member of the public can make a substantial contribution to such programs, as I documented 20% of the population of banded birds from North Carolina and 24% of the population of banded birds from Massachusetts, the latter being 1500 km from where they were banded. On a second level, the work contributes as an independent investigation. The data show how the many Gulf and Atlantic populations of skimmers interact. Although such an integrated perspective could be constructed if the many banding scientists shared the resighting data that they each received from the BBL, it is straightforward to compare the movements of the different populations using a single data set collected by one individual who used consistent methods at one geographic location. Moreover, by documenting record natal dispersal and gene flow between Gulf and Atlantic populations with photographs, and by highlighting the transformation of habitat caused by a Federal beach renourishment program that I observed firsthand during my surveys, I contribute local insights that were not obvious to the banding research scientists—all of whom are operating at a distance from this location. As such, this work advances understanding and contributes to the conservation of this imperiled species.

The research approach modeled here could be applied to investigate other species of birds [6] and non-avian species. A variety of non-avian species, ranging from fish [25], deer [26], butterflies [27], and sea lions [28] are being fitted with field readable markings with unique codes. For these species, too, a citizen scientist’s independent investigation could complement the work of the scientists tagging the individuals and provide insight into the biology of wide-ranging species. There is a growing body of evidence across a range of disciplines that demonstrates that citizen scientists can offer novel perspectives that can spur advances in a field [3,29,30].
